# Initiatives in biological research in Indian psychiatry

**DOI:** 10.4103/0019-5545.69222

**Published:** 2010-01

**Authors:** Amresh Shrivatava

**Affiliations:** Mental Health foundation of India, 209, Shivkrupa complex, Gokhale Road, Thane, Mumbai - 400 602, India

**Keywords:** Indian, research, biological psychiatry

## Abstract

Biological psychiatry is an exploratory science for mental health. These biological changes provide some explicit insight into the complex area of ‘brain-mind and behavior’. One major achievement of research in biological field is the finding to explain how biological factors cause changes in behavior. In India, we have a clear history of initiatives in research from a biological perspective, which goes back to 1958. In the last 61 years, this field has seen significant evolution, precision and effective utilization of contemporary technological advances. It is a matter of great pride to see that in spite of difficult times in terms of challenges of practice and services, administration, resource, funding and manpower the zest for research was very forthcoming. There was neither dedicated time nor any funding for conducting research. It came from the intellectual insight of our fore fathers in the field of mental health to gradually grow to the state of strategic education in research, training in research, international research collaborations and setting up of internationally accredited centers. During difficult economic conditions in the past, the hypothesis tested and conclusions derived have not been so important. It is more important how it was done, how it was made possible and how robust traditions were established. Almost an entire spectrum of biological research has been touched upon by Indian researchers. Some of these are electroconvulsive therapy, biological markers, neurocognition, neuroimaging, neuroendocrine, neurochemistry, electrophysiology and genetics. A lot has been published given the limited space in the Indian Journal of Psychiatry and other medical journals published in India. A large body of biological research conducted on Indian patients has also been published in International literature (which I prefer to call non-Indian journals). Newer research questions in biological psychiatry, keeping with trend of international standards are currently being investigated by the younger generation with great enthusiasm. What we have achieved so far is the foundation work in last 60 years. Our main challenge in development of biological psychiatry research in India remains resources in terms of manpower, funding and dedicated time for research psychiatrists. Developing basic sciences laboratories, discrete research questions, high quality methodology, and logistical support are some of the essentials. In the present time the culture of research has changed. It is specific and evidence-based. We have time-tested examples of International collaborative research. We need to get more resources, develop education, collaboration and effective leadership. In times to come, India will provide international leadership in basic and clinical biological psychiatry. There is hope.

## INTRODUCTION

Biological psychiatry is an exploratory science for mental health.[1] We now understand mental illnesses from a bio-psychosocial perspective, wherein biological, psychological and social factors combine in a unique, thus far unknown, way to cause a mental disorder.[2] Traditionally it has been the understanding that biological changes in human body lead to change in behavior. These biological changes provide some explicit insight into the complex area of ‘brain-mind and behavior’.[3,4] Psychiatric research has gone through the motion ‘Brainless minds’ to ‘Mindless brain’. We now understand more clearly the interconnection and integrated theory of several factors related to causation of mental disorders. A major achievement of research in biological field has been the findings to explain how does biological factors cause changes in behavior.[5] More recently, we also notice that biological research is involved in exploring how and what biological changes are caused by social, psychological and environmental factors, which affect mental health. Research in biological psychiatry has been a central area of research interests of Indian psychiatrists working in India as well as overseas. We have a clear history of initiatives in research from biological perspective, which goes back to 1958. In this brief review I plan to focus primarily on the studies that have attempted to find out biological causes of mental illnesses and biological changes occurring in mental illness. An important area of using psychopharmacology as a probe to understand the same question is not being covered in order to avoid duplication within this volume. This review does not discuss disease specific research and its findings in the field of biological psychiatry, which is covered in specific chapters covering several mental disorders in this volume. I will focus on development of the specialty, challenges present today and strategies to deal with them besides main researchers and their key research interest in an appendix.

## ACHIEVEMENTS

In last 61 years this field has seen a significant evolution, precision and effective utilization of contemporary technological advances.[5] It is a matter of great pride to see that in spite of difficult times in terms of challenges of practice and services, administration, resource, funding and manpower the zest for research has been very forthcoming. Some of the areas of research like ‘emotional stress’,[6] ‘cognition’,[7] ‘electro convulsive therapy’,[8,9] ‘psychophysiology’,[10,11] and ‘suicide,’[12] which were touched upon in 1950s and 60s still continue to elude researchers. Those days psychiatry was in the confines of mental hospitals, driven by treatment of severe mental disorder with very limited therapeutic tools. Research was not on the agenda. It was a passion. There was neither a dedicated time nor any funding for conducting research. It came from intellectual insight of our fore fathers in the field of mental health. It gradually grew to the state of strategic education in research, training in research, international research collaborations, settings and accreditation of centre’s from the World health organization, establishment of basic science laboratories, sophisticated neuroimaging, governmental collaborations and support and many other innovative initiatives, which speaks of about ‘leadership in biological psychiatry research’. This has been the single most important achievement in Indian Psychiatry.

It is not so much important what hypothesis was tested and what conclusions were derived. It is more important how it was done, how it was made possible and how traditions were established. A culture of research, much more sophisticated in contemporary medicine, was inherited by the younger generation. Biological research was conducted in mental hospitals, general hospitals, teaching hospitals, private clinics and in voluntary organizations. It continues to flourish with matching sophistication of developed world despite continuous problems of funding and lack of Governmental agenda.

## RESEARCH INTERESTS

The first generation Indian psychiatrists were trained in international institutions of repute. They possessed high level of insight into cultural and social roots of India and they were able to effectively combine these components in their research curiosity. Neurochemistry,[11,12] psychoendocrine,[13,14] immunoglobins,[15] body fluids,[16] electroencephalography,[17] electroconvulsive therapy,[18–21] neurotransmitters,[22] psychobiology,[23,24] cerebrospinal fluids,[25] neuropsychtric models and signs,[26,27] epilepsy,[28,29] neuro imaging,[30,31] plasma cortisole,[32] dexamethosone suppression test,[33,34] lithium,[35,36] serum chemistry,[37,38] metabolic effects,[39] teratogenicity,[40] cognition,[41,42] cannabis,[43,44] dermatographics,[45–47] experimental animal studies,[48] psychophysiology,[49] biological markers,[50] thyroid,[51] heritability and genetics,[52,53] cerebral dominance,[54] and yogic sciences[55] have been some of the major research interests of Indian psychiatrists across severe as well as common mental disorders published in the Indian journal of psychiatry. Some of these studies have achieved high citation in literature and some others still remain the key resource for new research. Interdisciplinary and inter-institutional collaborative research in neurosciences is a phenomenon of the past 15 years. All other work has been done by developing local resources, which speaks of an excellent stride and commitment for biological science.

What Indian psychiatry has witnessed in N.S. Vahia, V.N. Bagadia, A. Venkoba Rao, B.B. Sethi, S.M Channabavavanna and others, in terms of furthering Biological psychiatry, speaks of their exceptional leadership in difficult times. We hope that younger scientists will realize that it is not merely important to have a research initiative and peruse that, it is also important in the Indian context to create a conducive environment, resource, education and training for the generation next.[56] Psychiatrists from India working abroad have investigated almost similar research questions. A list of some of these is given in appendix.

## PUBLICATIONS

A lot has been published given the limited space in the Indian Journal of Psychiatry and other medical journals published in India. A large body of biological research conducted on Indian patients has also been published in International literature (which I prefer to call non-Indian journals). A substantial contribution has been made by Indian psychiatrists trained in India and working overseas. We are extremely proud of these excellent biological research inquiries explored by our own scholars at the international level. A number of them have acquired positions of distinction and acclaim in research. They are second to none and continue to provide vibrant leadership in International biological psychiatry research. A lot of work done in the field of biological psychiatry unfortunately has not been published. It remains confined to abstract books of annual and zonal conferences. A number of dissertations done during postgraduate education in psychiatry have remained unpublished. This is one area, not only in biological but psychiatric research in general, which needs significant improvement.[57] Training for writing skills and publication needs to become part of the mainstream psychiatric education.[58]

Key word and names of principal authors of biological psychiatric research published in Indian Journal of Psychiatry are Appendixes here for the purpose of readers and researchers benefit [Table 1].

**Table 1:** Indian author-publications in different fields of biological psychiatry in Indian journal of Psychiatry. (some of the volumes are unavailable, thus omission is regretted; any factual error is also regretted as it is purely coincidental omission)

Topic	Sub-topic	First author	Year of publication
ECT	Ectonus	Murti D.L.N. Rao	1958
	Electric and chemical convulsive	David J Impastato	1960
	Analysis of 5021 ECT	A.V.Shah	1962
	Four techniques	V. N Bagadia,	1962
	Memory and intelligence	H Narayan Murthy	1966
	Unilateral and bilateral	L. G Kiloh	1971
	ECG changes	R.K Jain	1976
	The efficacy of spaced v/s daily	D.K Deshmukh,	1980
	Propanidid	M.R Kulkarni,	1980
	ECT Phenothiazine Combination	A.K. Agarwal,	1985
	A review	G.D. Shukla	1989
	Althesin and thiopentone	R.K. Mahendru	1989
	Psychobiological frontiers	Chittaranjan Andrade	1990
	E.C.T a need of reappraisal	A.K. Agarwal	1990
	Seizure duration estimates	D.K Subbakrishna	1992
	Seizure duration	Chittaranjan Andrade	1993
	ECT induced EEG seizure	B.N Gangadhar	1993
	Physical morbidity	Prathap Tharyan,	1993
	Merits of EEG monitoring during ECT	K. Girish	2002
	Molecular mechanisms underlying electroconvulsive therapy-induced amnestic deficits: A decade of research	Chittaranjan Andrade	2008
Psychosomatics	Blood glucose	T.H.Seth	1958
Neuropsychiatry	EEG		1958
	The Neurology and bio-chemistry of behavior	Satya D Nand	1960
	Cerebral arteriosclerosis	A.K. Chatterjee	1965
	With temporal lobe epilepsy	G. D Shukla,	1981
	Cerebral laterality in schizophrenia	R.K. Gaur	1985
	Epileptic psychosis	Antony Fernandez	1988
	Neurological “Soft signs”	Alice Cherian	1989
	Eye movements	Ram Sharan	1990
	Neuroanatomical correlates	S. Sabhesan	1990
	Temporal lobe focus	Jambur Ananth,	1990
	Neurological abnormalities	K.S. Shaji,	1990
	Visual information processing deficits	C.V Ananthanarayanan,	1993
	Cerebral blood flow and metabolism	Roy J Mathew	1994
	neuropsychiatric model of psychosis	Shrivastava Amresh	1996
	Progressive multifocal leucoencephalopathy in AIDS	Pradeep Kumar	2006
	The limbic system	V RajMohan	2007
	Fronto-temporal dysfunction in schizophrenia: A selective review	John P John	2009
Neurochemistry	Body fluids	R. B Davis	1960
	Guaiacol Glycerol Ether (GGE)	C. H Mehta	1960
	Phosphate and creatinine excretion	P Subrahmanyan,	1962
	Acetyl methyl carbinol metabolism	Mc C. W Anderson	1962
	Functional hypoglycemia	Sankara A. Rao	1963
	Extra-cellular fluid volume	Harish Verma	1966
	Liver function tests, serum oxidation tests, serum ascorbic acid and copper B.S Sridhara Rama Rao, Levels		1970
	Blood histamine, histaminase levels and tissue sensitivity to histamine	R.K Sanyal	1970
	Metabolic defect	Sridhara B	1974
	N-Acetylneuraminic acid levels in the cerebrospinal fluid	B.S Sridhara	1975
	Excretion of 3,4-Dimethoxy Phenyl - Ethylamine	B.S Sridhar Rama Rao	1976
	ABO blood groups	Gurmeet Singh	1979
	Plasma and erythrocyte sodium, potassium	N Pradhan	1983
	Renal and extra-renal functions	V. N Puri	1984
	Serum lipids	T. K Mishra	1984
	Platelet MAO activity	A.K. Gupta,	1985
	Serum immunoglobulins	I.V.L. Narasimha Rao	1985
	Electrolyte profile	M.S. Bhatia	1987
	Renal function tests	Kuruvilla Mathew	1988
	Renal function	M.K. Dhar,	1988
	Phenylketonuria	HS Narayanan	1988
	Blood groups	P. Lakshmi Reddy	1988
	Rheumatoid factor		1988
	Platelet MAO activity	J.K. Trivedi,	1989
	Immunoglobulins and viral antibodies	S.C. Tiwari,	1990
	Platelet MAO activity	I Sharma	1990
	Pharmacokinetics	A.D. Bhatt	1991
	Platelet MAO	Indira Sharma	1991
	Effect of negative ion	A Chitra Andrade	1992
	Immunomodulator in the treatment of schizophrenia	S. Agarwal,	1992
	Serum acetylcholinesterase level	Nilesh Shah	1992
	Australia antigen (HBsAG)	S. Chaudhury,	1993
	Psychoimmunology and functional psychoses	S.C. Tiwari	1994
	Serum lipid profile	Sandeep Verma,	1999
	CSF amines and their metabolites in first episode drug NaÃve schizophrenic	Anand L	2002
	Evaluation of antioxidant deficit in schizophrenia	Gora Dadheech	2008
	oxidative stress and interrelationship of important antioxidants	Om Prakash Singh1	2008
Neurotransmitter	5 HT	K.S. Sachdev	1960
	Central dopamine and serotonin turnover	R.S. Pandey	1987
	Dopamine postsynaptic receptor	Chittaranjan Andrade	1990
	Single electroconvulsive shock and dopamine autoreceptors	B.N. Gangadhar	1990
	Serotonin and its metabolites	J.K Trivedi	1992
	Alpha - 2 noradrenergic and dopamine postsynaptic receptor functioning	Chittaranjan Andrade	1993
	Glucocorticoid receptor dysfunction	Aju Abraham	2003
	Platelet serotonin level	Devasis Ghosh	2008
Cognitive neurosciences	Cognitive effects of chronic bhang	A.K. Agrawal	1975
	Cannabis (Ganja) and cognition	A Venkoba Rao	1975
	Memory in depression	S Chandra	1982
	Cognitive dysfunction in depression	I Sharma,	1984
	Complex motor programming	Sumant Khanna	1987
	Cognitive disorder and depression	A. Venkoba Rao	1989
	Cognitive dysfunction in depression	Sajiv John	1992
	Luria - Nebraska neuropsychological battery	Alaka Nizamie	1992
	Cognitive functions in epileptic patients	M.R Nainian	1993
	Neurocognitive impairment in HIV infection	Tony Edwin	1999
	Schizophrenic Luria-Nebraska neuro-psychological battery	B.P. Mishra	2002
	Neuro-psychological profile of epilepsy	B.P. Mishra	2002
	Executive functions in depression	Rajul Tandon	2002
	Neurocognitive dysfunction in mood disorders	Brian P Moore	2003
	Executive functions in schizophrenia	S Sabhesan1	2005
	Neuropsychological impairment in bipolar affective disorder	Mubeen Taj	2005
	Neurocognitive function in women affected by the Bhopal gas disaster	RN Sahu	2005
	Cognitive dysfunction and associated factors in patients with chronic schizophrenia	Latha Srinivasan	2005
	Cognitive deficits in psychiatric disorders: Current status	JK Trivedi	2006
	Cognitive and emotional effects of renal transplantation	AA Pawar	2006
	Neuropsychological disposition and its impact on the executive functions and cognitive style in patients with obsessive-compulsive disorder	Sreemoyee Tarafder	2006
	Wisconsin card sorting test: Normative data and experience	Adarsh Kohli	2006
	The trail making test in India	Triptish Bhatia	2007
	Cognitive deficits in children of alcoholics	Melvin Chagas Silva	2007
	Neuropsychology of prefrontal cortex	Shazia Veqar Siddiqui	2008
Genetics	Schizophrenia-A genetic study	R Ponnudurai	1989
	Fragile Xq-27	S.K. Murthy	1989
	Genomic imprinting	Ratanendra Kumar	2000
	Genetics of schizophrenia - An overview	R Ponnudurai	2003
	The future of psychiatric genetics	Vishwajit L Nimgaonkar	2003
	Serotonin transporter gene polymorphism	Mushtaq A Margoob	2008
Clinical Research in biological psychiatry	Sedation threshold test	N S Vahia	1965
	Thyroid disorder	A.K. Chatterjee	1965
	Electroplexy Dinshaw	R. Doongaji	1966
	Electrically induced sleep	Kirpal Singh	1966
	Patterns in dreams	S.S. Nathawat	1973
	Family study of atypical lymphocytes		1973
	Teratogenic study	N Sethi	1974
	Buccal and dermatoglyphic studies	S.S Agarwal	1975
	Habitual use of cannabis indica	V.N Bagadia	1976
	Palm prints	G Eswaraiah	1978
	Dermatoglyphics	R.S Balgiri	1978
	Tetratogenic effects	N Sethi	1980
	Negative symptoms	Santosh Chaturvedi	1985
	Work performance of schizophrenic	P.S. Gopinath	1985
	Season of birth	Rakesh Kumar Jangid	1989
	Dermatoglyphics	H.P. Jhingan	1989
	Dermatoglyphics	H.P. Jhingan	1990
	Finger pulse volume	P Bharathi	1992
	Finger - pulse volume during Co_2_ induced panic states	P Bharathi	1992
	Alcohol dependence: biological and clinical correlates	Pratima Murthy	2003
	Neurobiological basis of ganser syndrome	Daniel Ouyang	2003
	Sleep disorders in children with attention-deficit hyperactivity disorder	Subhash C Bhargava	2005
	Impact of vocational rehabilitation on social functioning, cognitive functioning, and psychopathology in patients with chronic schizophrenia	PN Suresh Kumar	2008
	Neurobiology of alzheimer’s disease	E Mohandas	2009
Neuophysiology, Psychophysiology and electrophysiology	Emotional stress		1960
	Autonomic response	Abraham Verghese	1970
	Eosinophil rhythm	Parvathi S Devi	1971
	Concepts of patanjali	N. S Vahia	1973
	Skin conductance responses	P. K Biswas	1981
	EMG bio-feedback	M. T Gada	1984
	Psychobiology of depression	M. T Gada	1987
	Psychobiology of suicide	A.Venkoba Rao	1987
	Electrocardiographic changes	S Haque	1988
	E.E.G abnormality	Alice Cherian	1990
	EMG biofeedback	D. Sargunaraj	1990
	EMG biofeedback	D Sargunaraj	1991
	Efficacy of meditation	Vihang N Vahia	1993
	Electromyograph feedback	A Abraham	1994
	Biology of addictions	Desai NG	1995
	P300 event related potential	R Shukla,	2000
	P 300 event related potential	R Singh	2000
	EEG alpha coherence and psychopathological dimensions	John P. John	2002
	Spirometry and airway reactivity	R.B Galgali	2002
	EEG fractal dimension and spectral	Jagadisha	2003
	REM sleep latency and neurocognitive dysfunction in schizophrenia	Mrinmay Das	2005
Psychoneuroendocrine	Dexamenthasone	S Kumar	1970
	Plasma cortisol	Abraham Verghese1	1973
	Pineal gland responses	S Parvathi Devi	1976
	Pineal gland RNA	N Hariharasubrmanian	1976
	Thyroid function	G.C Boral	1980
	Of pinel gland in clinical cases of psychological stress	P.M Singh	1980
	Pineal response to lithium	S Parvathi Devi	1982
	Dexamethasone suppression test	R. Ghulam	1985
	Psychoendocrinology and behavior	G.C. Boral	1986
	Dexamethasone suppression test	M.Saikumar Reddy	1986
	Serum prolactin levels	K. Kuruvilla	1986
	Dexamenthasone suppression test	G. Prasad Rao	1987
	Dexamethasone supression test	S.L. Varma	1987
	Dopamine related hormone levels in acute schizophrenia	S.B. Chatterjee	1988
	Adrenocortical dysfunction in depression	KC Gurnani	1988
	Serum prolactin	P. Tharyan	1988
	Dexamethasone supperssion test	S. Chaudhury	1989
	DST	A. Agarwal	1989
	Post dexamethasone plasma cortisol	R. Ghulam	1990
	ACTH and the dexamethasone suppression test	Ashok Kumar Jainer	1992
	Multiple endocrine responses to clonidine	Sumant Khanna	1992
	Concentrations of homovanillic acid and gonadal hormones	S.L Gong	1993
	Serum prolactin level	Amresh Shrivastava	2000
	Thyroid hormones	Jalaj Saxena	2000
	Is oestrogen a biological neuroleptic?	Subhagata Chattopadyay	2003
Neuroimaging	Structural changes in the brain in schizophrenia a computed tomographic study	S.K. Jayaswal	1987
	ECT and T2 relaxometry: A static walter proton magnetic resonance imaging study	K Girish	2001
	Reduced caudate volume in never-treated schizophrenia	Ganesan Venkatasubramanian	2003
	Enlargement of the third ventricle in affective disorders	Rano Bhadoria	2003
	Regional brain metabolism in schizophrenia: An FDG-PET study	R. Seethalakshmi	2006
	Study of childhood onset schizophrenia (COS) using SPECT and neuropsychological assessment	Savita Malhotra	2006
	MRI T 2 relaxometry of brain regions and cognitive dysfunction following electroconvulsive therapy	Girish Kunigiri1	2007
Therapeutics	Lithium and adrenal cortex	S Parvathi Devi	1973
	Lithium and kidney	Venkoba Rao	1981
	Lithium and renal functions	N. Sethi	1987
	24 hour serum lithium level	K. Kuruvilla	1989
	Lithium psychiatry	S. K Khandelwal	1991
	Bioavailability of lithium carbonate	S.K Tripathi	1993
	Long term effects of lithium	Baljinder Singh	2000
	Lithium toxicity	Ratanendra Kumar	2001
Conceptual, Thematic, Educational and Research	Research training in psychiatry	B.B. Sethi	1968
	W. H. O. collaborating centre for psychopharmacology		1974
	Teaching of psychopharmacology	J.V. Ananth	1976
	Handbook of biological psychiatry		1980
	Research in psychiatric genetics in India	R. Srinivasa Murthy	1983
	Psychiatric research	S.M. Channabasavanna	1987
	International collaborations in psychiatric research	S.M. Channabasavanna	1988
	Post partum psychiatric syndromes: Are they biologically determined?	Shiv Gautam	1989
	National workshop on “ECT: Priorities in research and practice”		1989
	The practice of ECT in India: Issues relating to the administration of ECT	A.K. Agarwal,	1992
	The practice of ECT In India: II. The practical administration of ECT	Chittaranjan Andrade	1993
	Do indian researchers read indian research?	Chittaranjan Andrade	1994
	Evidence based medicine in psychiatry	J.K. Trivedi	2000
	Clinical methods in psychiatry	Mukul Sharma	2000
	Evidence-based psychiatry: A distant dream?	N.G. Desai	2000
	Your research project	J.S. Srivastava	2001
	Research in biological psychiatry in India	V. Palaniappun	2002
	Research endeavors in child and adolescent psychiatry in indian	Shoba Srinath	2002
	Practice of ECT in India		2002
	Mind in indian philosophy	A. Venkoba Rao	2002
	Glimpses of neurobiological underpinnings of bipolar disorder		2003
	Publication of mental health research from poor income countries: Resolving the information divide!		
	Why ‘publish or perish’? Why not ‘publish and prosper’? Perspectives from developing countries	Nimesh G. Desai	2005
	Social origins, biological treatments	Vikram Patel	2005
	Mild cognitive impairment: The dilemma	Charles Pinto	2009

Significant events in growth and development of biological psychiatry research

1. Establishment of phychophysiological and clinical psychopharmacology department at the K.E.M Hospital, Mumbai.
2. Establishment of the National Institute of Mental Health and Neurosciences, Bangalore and several departments in clinical and experimental studies in Neurochemistry, Genetics and Neuroimaging.
3. Establishment of WHO collaborative center at the K.Gs medical college, Lucknow.
4. Establishment of WHO collaborative center at the K.E.M Hospital Mumbai.
5. Biological psychiatry unit at the Madras Medical College, Chennai.
6. Neurochemistry unit at CMC Vellore.
7. Department of Electroencephalographic studies at the Central Institute, Ranchi.
8. Indian psychiatric Society’s institution of an ‘oration award’ for excellence in Biological Psychiatry Research in India for psychiatrists under 40 years called ‘Tilak venkoba Rao Oration’.
9. ICMR Center of Research in Geriatric Psychiatry at Madurai.
10. ICMR, Ministry of Health, Government of India research initiatives in advanced neurosciences.
11. Department of Science and Technology’s initiative of research in neurosciences.
12. Establishment of four ‘centers of excellence’ in research and treatment of addiction psychiatry.
13. Department of addiction psychiatry at the All India Institute of Medical Sciences, New Delhi.
14. Publication of first Handbook of biological psychiatry.
15. International Biological psychiatry workshop at Bangalore.
16. Research training in Biological psychiatry at several centers in India.
17. WHO-ICMR Training workshop in Biological psychiatry and psychopharmacology for south East Asian countries at KG’ Medical College, Lucknow.
18. Publication of Handbook of Biological Psychiatry Research from NIMHANS, Bangalore.
19. Indian Psychiatric Society’s initiative of establishing ‘specialty Section on Biological psychiatry’.
20. A number of workshops and training courses organized by the Indian Psychiatric Society.
21. World Psychiatric Association’s Section meeting of Biological psychiatry: ‘International Convention of Biological psychiatry’ Mumbai, 1996.
22. Inaugural symposium of the new section of the World Psychiatric Association’s section on Psychoneuroendocrinology, during Annual national Conference of the Indian psychiatric Society, Jaipur.
23. Indo-US initiative for research and education in Genetics.

### Current research questions in Biological Psychiatry in India

There are two contemporary theories of mental illness. According to one theory, these disorders are biological in origin and their origin, management, course, outcome and preventive strategies are almost across cultures, regions and economic class.[59] The second theory claims that these disorders only have a biological dimension.[60] The biological factors in their own right are neither enough do they determine origin, course, management, outcome and prevention.[61] Heritability in itself does not cause diseases and socio-economic conditions do not necessarily manifest as psychiatric disorders.[62,63] Though there is consensus for the ‘bio-psychosocial’ model for psychiatric disorders, the pathways for manifestation of symptom, diagnostic criteria, outcome measures and prevention of mental disorders based upon this model are poorly understood.[64] It has been repeatedly demonstrated that psychiatric disorders are culturally influenced and some times culture-specific.[65]

The high quality research arising from human genomics to explain the complexity of gene-environment interaction in expression of symptoms and their response to treatment has not given specific findings as yet.[66] The field of pharmacogenetics and microbiology in mental health is also in its infancy. Indian culture, societal structure, social variability, regional heterogeneity and economic disparities are more than obvious. Biological research needs to target how social conditions influence mental illnesses. A lot of work is needed in the field of ethno-psychopharmacology and pharmacogenetics to understand efficacy and side effects of psychotropics used. Another important area to explore is the complexity of brain-mind and behavior from neurobiological perspectives.[67] Several unfortunate conditions like trauma, natural disaster, violence and abuse continue to influence manifestation and outcome of psychiatric disorders. Little attention has been paid to this important area from biological point of view.[68–70] Impact of stress on medical disorders, exploring dimensions of gene-environment interaction, biological probes for changing behavior patterns are some of the priorities.[71] Finally, nothing is more important than prevention of metal illnesses. So far this area has remained within the confines of clinical public health presuming that biological psychiatry has little to offer in terms of prevention, which is not the fact. The current research on ultra high-risk individuals, prodormal phase and early psychosis has demonstrated that early intervention from biological therapies can be successful for prevention.[72] At the minimum, it is useful in limiting the severity and disability of psychosis. Similarly, we need to urgently understand biological markers of diagnostic groups, and response to treatment across all mental disorders. A biological basis of risk and protective factors is a significant question in suicidology research. Neurobiology of brain development and factors interfering with it in the pathway of brain maturation are part of another area of priority. ‘Changing behavior’ may have a biological answer to what would be helpful to adjunct cultural, social, psychological, spiritual and religious ‘therapies’.

### Challenges and strategies

What we have achieved so far is the foundation work in last 61 years. Our leaders have done enough in preparing the runway and it’s now time to take off for the younger generation. It was voiced from our organization’s platform more than a decade back that reframe ‘capabilities we have and resources we need’.[73]

Our main challenge for development of biological psychiatry research in India continues to be resources in terms of manpower, funding and dedicated time for research psychiatrists. Developing basic sciences laboratories, discrete research questions, high quality methodology, and logistical support are some of the essentials. In the present time the culture of research has changed. It is specific and evidence-based. There in no space for vague questions. Our post-graduation courses are required to have clear deliverables and doable objectives for education in research. Courses like advanced training, fellowship schemes, training in research methodology, statistics, designs, grantmanship, critical appraisal, writing skills and art of publications are some of the fields which need urgent attention. The initiative needs to come from universities, teaching institutions, research institutes, local governments and professional bodies.

The Indian Psychiatric Society in particular needs to do more to become role model for other institutions. There has to be some mechanism within this organization and funding bodies. A good example is the research initiative from the World Psychiatric Association, which has developed effective liaison with few reputed universities for research grants and education in research. This model is effective. We are growing in economic terms. I personally think it is possible that small steps will go long way. An annual grant of even Rs 10,000 from the IPS will be encouraging. We need to develop a culture of grantmanship in our students. Research in Biological area is not possible without large amount of money. Non-governmental bodies are available at national and international level for this purpose. Another key strategy is to develop skills in national and international collaboration in interdisciplinary research. We need to understand that education, research and clinical services are inter-related. A rich, honest and respectful environment will attract a lot of psychiatrists who lack opportunities. Networking with in the city and country is necessary. A divide between institutional and non-institutional psychiatrist is not helpful. We need an integrated approach, which is inclusive, and reflective of our population e.g. the first episode studies being performed in tertiary psychiatric institutions are less helpful. We loose the clinical population by our lack of initiative. The next strategy is to do research on a broader canvas and sustain it over the required period of time. Cross-sectional research is less qualitative than prospective longitudinal ones. Therefore, to sustain the research interest is extremely important the cost of such work needs to be built-in to the project. Quite a few inquiries die beyond pilot studies because of one reason or the other and most commonly it is the transfer of posting in service conditions. Developing strong teamwork will certainly be helpful. Last but not the least is need for focus for researchers. It is the specificity which will provide them their niche, recognition and rewards, re-enforcing future work. Popularizing and marketing your research effectively is another academic exercise. Its part of several universities’ agenda for continued professional development program. We need to believe in it and bring it into practice.

### International collaborations in biological Psychiatry

In times of globalization, world has become all-inclusive. Research from India has gained significantly. In practical terms, we need to enhance our quality and reputation at the international level in the field of biological psychiatry. Work, which has been done, will be popular with citation rate. Until now only a select group of researchers are well cited [Table 2] and this needs to change. International collaborations are very effective. Just to mention some work done in India with international collaborations, e.g. by Dilip Jeste on Tardive dyskinesia,[74] Neuroimaging by M.S. Keshavan.[75] Microbiology by Sahebrao Mahadik,[76] and Genetics by V.S. Nimgaokar[77] and several others in the field are successful examples. We need to catch up in this field. Psychiatric education needs to provide autonomy of thinking, practice and innovation.[78]

**Table 2:** Some of the significant research interests of Indian investigators published in International journals

ECT	Experimental designs	Andrade CA
	Use in Parkinson’s	Goswami U
	Cochrane database	Tharyan P
Schizophrenia	Acute brief psychosis	Collins PY, Wig NN,
	Neurochemistry	Arvindakshan M
	Cross cultural emotional processing	Habel U, Gur RC
	Membrane abnormality in Basal ganglia	Jayakumar PN,
	DST	Joseph S
	Viral antibodies	Srikanth S,
	Genetics	Vaswani M
	Genetis and heritability	Verma R
Social phobia	psychobiology	Chatterjee S
Depression	Serum cholesterol	Das PP
	Bereitschafts potential	Khanna S
	Evoked potential	Khanna S
	Neuropsychiatric signs	Nizamie SH
	CSF Amines	Reddy PL
	Erythrocyte membrane	Reddy PL
	P300	Santosh PJ
Mania	Platelet serotonin receptor	Velayudhan A
Markers	DST	Goswami U
	P300	Murthy PJ
	CSF 5HT	Narayan M
	CSF enzymes with haloperidol challenge	Pai BN
	CSF amines	Reddy PL
Prefrontal cortex	intracellular calcium	Jagadeesh SR
Cognition	ECS induced antrgrade amnesia	Joseph J
	Experimental design	Kumar KB
	Retrieval in experimental animal design	Kumar KB
Neuropsychiatry	Neuroleptic induced dystonia	Khanna R
	Klein levine syndrome	Malhotra S
	Acute intermittent porphyria	Santosh PJ
OCD	Frontal lobe dysfunction	Khanna S
	neuroendocrine	Khanna S
	Viral antibodies in CSF	Khanna S
Alcoholism	Apolipoproteins and lipids	Meera V
	Biochemical measures	Vaswani M
Experimental design	poststartle activity	Munonyedi US
Dementia	CSF Zinc	Sahu RN
Conceptual and thematic	Biological psychiatry in India	Sethi BB

[N. B. This is not a complete list but only an example for reference. This does not mean ‘selected’ or ‘preferred’]

## CONCLUSION

Biological Psychiatry research in India has grown to some degree. Indian conditions had not been conductive to this research but the commitment and leadership in psychiatric research is commendable. Very interesting and locally pertinent research questions have been addressed in the 60 years. A lot has been published in Indian Journal of Psychiatry and in International journals of high impact. However, we are much away from where we need to be. We need to get resources, develop education in biological research, and develop effective leadership and collaboration. We need to remind ourselves of our responsibilities, be mindful of central focus in research questions and use international culture and techniques effectively. We need to address some of the culturally relevant questions. It is possible, it is doable and it must be done. Most of what I have written sounds ‘political’ but that is how it is at this point of time.

N.B: Any omission of facts on Indian research in Biological Psychiatry in this review is purely ‘unawareness’ which is inadvertently committed and regretted.
